# Perspectives on SRS Imaging of Nanoparticles

**DOI:** 10.1021/accountsmr.3c00100

**Published:** 2023-07-31

**Authors:** Lingyan Shi, Hongje Jang

**Affiliations:** Shu Chien-Gene Lay Department of Bioengineering, UC San Diego, La Jolla, California 92093, United States

## Introduction

Stimulated Raman scattering (SRS) microscopy is a nonlinear optical imaging method. It has emerged as a powerful technology for quantitative imaging of nanoparticles (NPs) in diverse material systems.^1^ This Viewpoint presents an overview of recent advancements of SRS microscopy in this field, highlighting its principles and advantages.

**Figure 1:**
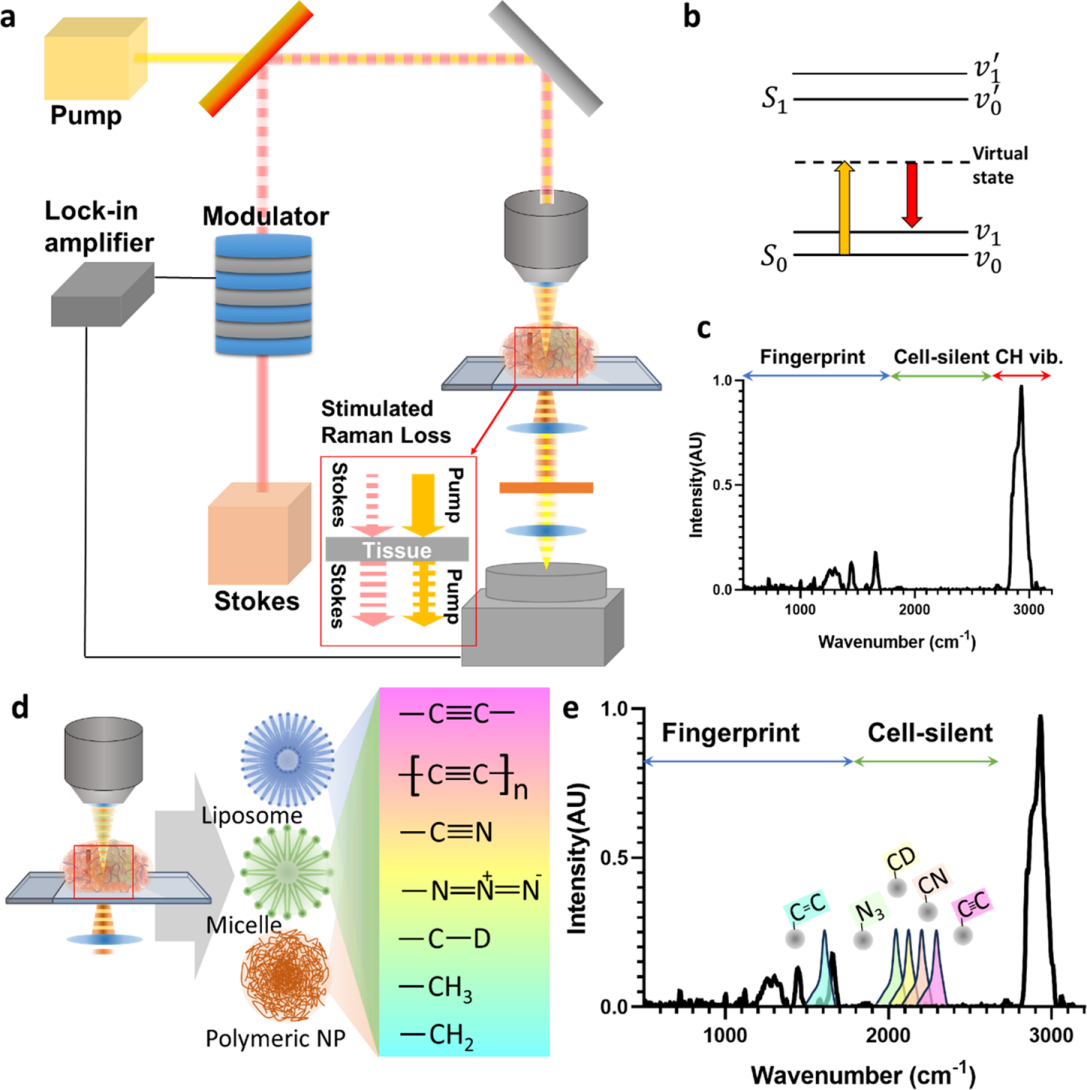
Mechanism of SRS. (a) Setup of SRS microscopy. Two synchronized laser beams, the pump and Stokes beams, are used. (b) Process of SRS. (c) SRS signals are detected in the fingerprint region and CH vibration region in the Raman spectrum. (d) Liposomes, micelles, and polymeric nanoparticles can have various functional groups that can be detected by SRS microscopy. (e) Each different functional group has different vibrational energies, and some of them have unique energy levels that cannot be detected from natural biomolecules.

SRS microscopy uses two synchronized laser beams, the pump and Stokes beams (Figure 1a,b). The SRS signal is generated when the energy difference between these two beams matches the vibrational energy of specific molecular bonds, for example, −CH_3_ and −CH_2_ vibration of protein and lipid in CH vibration region (Figure 1c), which enables selective imaging of chemical bonds.^1^ By detecting the vibrational signatures of molecules, SRS microscopy allows for the visualization and analysis of NPs within specimens without additional labels.^2^

SRS microscopy offers several distinct advantages for quantitative imaging. First, it is label-free, which eliminates the need for exogenous probes or dyes, thus preserving the native properties of NPs and the surrounding material.^3^ This allows for the direct observation of NPs in their native environment. Second, the SRS signal is linearly correlated with molecular concentration, facilitating quantitative chemical imaging. Third, SRS imaging is generally fast, allowing for the acquisition of large-area images with minimal sample degradation.^4^ This is particularly advantageous for studying dynamic processes, such as diffusion, aggregation, and dissolution of NPs. In addition, SRS microscopy offers high spatial resolution down to submicrometer scale.

## Applications of SRS Microscopy in Nanoparticle Characterization

SRS microscopy has found extensive applications in biological studies. It enables the visualization of NP uptake and distribution within cells and tissues, offering insights into their mechanisms and potential biomedical applications. It has also been applied to investigate NP-based drug delivery systems, providing valuable information on drug release kinetics and targeting efficiency. Studies demonstrated the utility of bioorthogonal chemical bonds for imaging NPs and gaining insights into their cellular fate.^5^ Polymeric NPs have gained attention as promising carriers for targeted drug delivery, but their nanoscale size poses challenges in understanding their uptake and localization within cells. To address this, researchers employed small chemical labels attached to poly(lactic acid-*co*-glycolic acid) (PLGA) to create NPs amenable for SRS imaging. By introducing alkyne signatures in modified PLGA terpolymers, researchers imaged both deuterium and alkyne-labeled NPs in primary rat microglia, and alkyne-labeled NPs in mouse cortical tissue ex vivo.^6^ Immunohistochemical analysis confirmed the localization of NPs in microglia, indicating their potential for targeted therapeutic delivery to these cells.^7^ This approach offers a valuable means to comprehensively characterize the uptake, spatial distribution, and cellular interactions of NPs. By leveraging bioorthogonal chemical bonds and SRS microscopy, one can gain critical insights into the behavior of polymeric NPs in biological systems, addressing safety concerns and advancing the development of efficient drug delivery strategies.

SRS microscopy has also been applied to the field of nanomaterials, on characterizing the composition and distribution of NPs in complex matrices.^8^ From studying plasmonic NPs in energy devices to investigating catalytic NPs in heterogeneous systems, SRS microscopy has provided crucial information on their spatial distributions, chemical compositions, and surface interactions.

## Future Perspectives

While SRS microscopy holds great promise for quantitative imaging of NPs and has been recognized as a valuable and effective imaging technique for in vitro and vivo studies of nanocarriers in cell and tissue,^1^ several challenges remain.

The first challenge is the spectral overlap between Raman signals of NPs and background molecules, which can hinder accurate quantification of NPs. Advanced spectral unmixing algorithms and data analysis techniques are being developed to address this issue.^3^

A second challenge is related to the concerns of biocompatibility and toxicity of NPs for clinics. Polymeric and liposomal carriers (Figure 1d,e) are made of either polymers or lipid-based materials that are widely used in clinic due to their biocompatibility.^9^ FDA approved PLGA is also an attractive material for clinics. As the clinical potential is realized, concerns regarding nanocarriers’ toxicity become important.^10^

SRS allows for direct imaging of NPs in cell and tissue, which is important for studying the uptake and degradation of NPs. The uptake and degradation times are essential factors to determine NPs’ potential as intracellular drug delivery vehicles,^11^ and they are dependent on the type of cells, as well as on the properties of NPs including the size, surface properties, and zeta-potential.^12^

SRS imaging inherently is a nondestructive and label-free imaging method that does not need bulky labels. Image contrast of NPs in tissues has been shown to be enhanced by label-free SRS imaging.^13^ On the other hand, deuterium and alkyne tags have also been used for imaging NPs by probing the carbon-deuterium or alkyne bonds.^2^ Multicolor SRS images of NP in cells have been achieved by using deuterium labeling. These capabilities highlight the future potentially broad applications of SRS imaging in visualizing cellular NP dynamics. In clinical applications, real-time SRS imaging of nanocarriers’ distribution in living organisms can be realized.

Further advancements in SRS imaging technologies, such as improvements in sensitivity, signal-to-noise ratio, and resolution, will enhance the capabilities for precisely localizing and characterizing NPs. The utilization of advanced digital analysis algorithms, including PRM-SRS microscopy for hyperspectral image analysis^15^ and A-PoD for converting diffraction-limited images into super-resolved ones,^16^ enables the detection of nanoscopic molecular heterogeneity in tissues, cells, and even in the subcellular organelles such as mitochondria, endosomes, lysosomes, and lipid droplets. This super-resolution chemical bond imaging can also map out the spatial temporal interactions between NPs and organelles in live cells *in situ*, which potentially opens a new research direction for quantitative imaging of their mutual effect. Additionally, the integration of SRS microscopy with other imaging modalities, such as fluorescence microscopy and electron microscopy, could provide complementary information, enabling a more comprehensive understanding of NP properties and its dynamics through cell membranes and cytoplasm.

## Conclusion

In summary, SRS microscopy represents a powerful technology for quantitative imaging of NPs in various contexts. With its label-free and high-resolution imaging capabilities, SRS microscopy offers unique advantages in NP characterization, enabling the visualization and analysis of NPs in their native environments. As the field continues to advance, SRS microscopy holds immense potential for improving our understanding of NP behavior, interactions, and for broad applications in biomedical or clinical studies.
